# Supplementary material for: Electrochemical Biosensor for Detection of the CYP2C19*2 Allele Based on Exonuclease Ⅲ
Source: Micromachines (Basel). 2023 Feb 25;14(3):541. doi: 10.3390/mi14030541 (PMC10053321; doi:10.3390/mi14030541)
Supplement: Supplementary file 1 [file micromachines-14-00541-s001.zip › Marked Supplementary Material.pdf]

Supporting Information for Electrochemical Biosensor for Detection of the CYP2C19*2 Allele Based on Exonuclease Ⅲ

Siling Chen [1], Rongjun Yu [1], Ying Li [2], Jiangling Wu [1], Jingfu Qiu [2], Xinyi Huang [3,*] and Jianjiang Xue [1,*]

[1] Department of Clinical Laboratory, University-Town Hospital of Chongqing Medical University, Chongqing 401331, China; chensiling1203@163.com (S.C.); yurongjun@hospital.cqmu.edu.cn (R.Y.); wujiangling@hosipital.cqmu.edu.cn (J.W.)

[2] School of Public Health and Management, Chongqing Medical University, Chongqing 400016, China; liiiyiii@stu.cqmu.edu.cn (Y.L.); jfqiu@126.com (J.Q.)

[3] Department of Clinical Laboratory, First Affiliated Hospital of Guangxi University of Chinese Medicine, Nanning 530023, China

* Correspondence: xinyihuang1210@163.com (X.H.); jianjiangxue@163.com (J.X.)

S1. Buffers

The buffers and solutions involved in this experiment were as follows: DNA preparation solution (20 mM Tris-HCl containing 5 mM KCl, 140 mM NaCl, 1 mM MgCl2 and 1 mM CaCl2, pH 7.4), TCEP buffer (140 mM NaCl, 5mM KCl, 20 mM Tris-HCl and 10 mM TCEP, pH 7.4), washing buffer (Tris buffer including 20 mM Tris-HCl, 100 mM NaCl, 5 mM MgCl2, pH 7.4 and Tris buffer including 20 mM Tris-HCl, 100 mM NaCl, 5 mM MgCl2, 0.05% Tween-20, pH 7.4), detection buffer (0.1M PBS，pH 7.4)). The buffer for both cyclic voltammetry (CV) and electrochemical impedance spectroscopy (EIS) consisted of 0.1 M PBS, 0.1 M KCl and 10 mM $[Fe(CN)_6]^{3-/4-}$ (pH 7.4). Ultrapure distilled water (18.2 MΩ/cm) obtained from a Millipore Mill-Q purification system was used for all solution preparation.

Table S1. Synthetic oligonucleotide sequences used in this study.

**Nucleotide name**	**Sequence (5′®3′)**
Target DNA (tDNA)	GATTATTTCCCAGGAACCCATAACAAATTA
Hairpin capture probe (HC)	SH-AAAAAAAAAAAAAAACCCAGGAACC<br>CATAAACACTTACAGACTGATCACATTA<br>TGGGTTCCTGGGAAATAATC
Labeled signal probe (LP)	TACTCCCCCAGGTGCAATCATGTGATCAGTCT<br>GTAAGTGTTTATG-SH
Auxiliary probe (AP)	TGATTGCACCTGGGGGAGTACATAAACACTT<br>ACAGACTGATCACA
Single-base mismatch target (1-MT)	GATTATTTCCCTGGAACCCATAACAAATTA
Two-base mismatch target (2-MT)	GATTATTACCCTGGAACCCATAACAAATTA
non-complementary DNA(NC)	CACTAAGGGCAATTAACCCATAACAAATTA

Table S2. Analytical performance compared with other methods of *CYP2C19*2* gene detection.

Methods	Detection methods	linear range	LOD	advantages and disadvantages	Reference
Electrochemical biosensor	CV or amperometry	—	—	The results are accurate but the detection range is unclear	[4]
Sandwich-type electrochemical biosensor	Amperometry	1 fM -50 nM	0.33 fM	Wide detection range but Hazardous chemicals are needed	[5]

Sandwich-type electrochemical biosensor based on DNAzyme and calixarene	DPV	0.01fM-100pM	13.49aM	Lower detection limit but time-consuming	This study

Table S3. Determination of target DNA in human serum samples (n=3) with the proposed biosensor.

Sample	Added target DNA (pM)	Found (pM)	Recovery (%)	RSD (%)
1	0	0	-	-
2	0.1	0.1012	101.20	2.70
3	1	1.0046	100.46	2.40
4	10	9.667	96.67	0.51
5	50	53.9884	107.98	0.39

1. Liu, G. Single-Nucleotide Polymorphism Genotyping Using a Novel Multiplexed Electrochemical Biosensor with Nonfoulingsurface. *Biosensors and Bioelectronics* **2013**, 6.
2. Zhang, C.; He, J.; Zhang, Y.; Chen, J.; Zhao, Y.; Niu, Y.; Yu, C. Cerium Dioxide-Doped Carboxyl Fullerene as Novel Nanoprobe and Catalyst in Electrochemical Biosensor for Amperometric Detection of the CYP2C19*2 Allele in Human Serum. *Biosensors and Bioelectronics* **2018**, *102*, 94–100, doi:10.1016/j.bios.2017.11.014.
